# Association of *TGFB1* rs1800469 and *BCMO1* rs6564851 with coronary heart disease and *IL1B* rs16944 with all-cause mortality in men from the Northern Ireland PRIME study

**DOI:** 10.1371/journal.pone.0273333

**Published:** 2022-08-22

**Authors:** Rachel E. Mooney, Gerry J. Linden, Lewis Winning, Katie Linden, Frank Kee, Pascal P. McKeown, Jayne V. Woodside, Christopher C. Patterson, Gareth J. McKay

**Affiliations:** 1 Centre for Public Health, Queen’s University Belfast, Belfast, United Kingdom; 2 Dublin Dental University Hospital, Trinity College Dublin, Dublin, Ireland; Centro Cardiologico Monzino, ITALY

## Abstract

**Background:**

Historically, high levels of morbidity and mortality have been associated with cardiovascular disease in the Northern Ireland population. Previously reported associations between single nucleotide polymorphisms (SNPs) and cardiovascular disease within other populations have not always been consistent.

**Objective:**

To investigate associations between 33 SNPs with fatal or non-fatal incident coronary heart disease (CHD) events and all-cause mortality in the Northern Irish participants of the Prospective Epidemiological Study of Myocardial Infarction (PRIME).

**Method:**

Phase 2 of the PRIME study prospectively evaluated 2,010 men aged 58–74 years in Northern Ireland for more than 10 years for incident CHD events (myocardial infarction, percutaneous coronary intervention, coronary artery bypass, and cardiac death) and more than 15 years for all-cause mortality. SNPs previously reported in association with cardiovascular outcomes were evaluated against incident CHD events and all-cause mortality using Cox’s proportional hazards models adjusted for established cardiovascular disease risk factors.

**Results:**

During the follow-up period, 177 incident CHD events were recorded, and 821 men died. Both *BCMO1* rs6564851 (Hazard ratio [HR] = 0.76; 95% confidence intervals [CI]: 0.60–0.96; P = 0.02) and *TGFB1* rs1800469 (HR = 1.30; CI: 1.02–1.65; P = 0.04) were significantly associated with incident CHD events in adjusted models. Only *IL1B* rs16944 was significantly associated with all-cause mortality (HR = 1.18; CI: 1.05–1.33; P = 0.005). No associations remained significant following Bonferonni correction for multiple testing.

**Conclusion:**

We report a novel association between *BCMO1* rs6564851 and risk of incident CHD events. In addition, *TGFB1* rs1800469 and *IL1B* rs16944 were associated with the risk of incident CHD events and all-cause mortality outcomes respectively, supporting previously reported associations.

## Introduction

Cardiovascular disease (CVD) is the leading cause of global mortality according to the World Health Organisation, accounting for approximately 17.9 million deaths in 2016 [1]. In 2017, 94% of CHD deaths in NI were attributed to modifiable risk factors [2], including diet [3], obesity [4] and smoking [5]. Non-modifiable risk factors including age [6], male sex [7], ethnicity [8, 9] and socioeconomic status [10] have also been reported to influence CHD risk. Given the multifactorial nature of the risk associated with CHD, the genetic influences of single nucleotide polymorphisms (SNP) as risk factors for CHD is less clear. With rapid whole genome sequencing now possible through technological advances [11], genetic testing offers potential for diagnosis, patient risk stratification and management decisions [12]. Polygenic risk scoring may prove beneficial, with almost 8% of the population reported to have a 3-fold increased CHD polygenic risk [13]. Genetic testing is currently recommended for some cardiac conditions, such as long QT syndrome and hypertrophic cardiomyopathy but the potential application of genetic testing across CVD in general, remains largely unexplored [14].

The Prospective Epidemiological Study of Myocardial Infarction (PRIME) was established to prospectively study risk factors of CHD in Northern Irish and French men [15]. Among the Northern Irish PRIME participants, 33 SNPs from 23 genes were genotyped, selected on the basis of previously reported associations with CHD or CHD risk factors. These SNPs were based on multi-functional gene sets, including 10 SNPs from eight genes encoding interleukins or interleukin receptors. Interleukins play an important role in inflammation [16], a known risk factor for CHD [17], with several previously reported in association with CHD—*IL6* rs1800795 [18], *IL6R* rs2228145 [19], *IL10* rs1800872 [20] and *IL17A* rs2275913 [21].

Furthermore, additional candidate SNPs from other genes with previous evidence of association with CHD or CHD risk factors were also evaluated. These included SNPs from vitamin D receptor (VDR) [22], RANK ligand [23], transforming growth factor beta [24], toll-like receptor 4 [25], cytochrome c oxidase subunit II [26], S100 calcium binding protein A8 [27], N-acetyltransferase 2 [28], beta-carotene 15,15’-monooxygenase 1 [29], solute carrier family 23 member 1 [30], CD36 molecule [31], glycosyltransferase 6 domain containing 1 [32], and fibronectin [33]. Several of these candidate SNPs were chosen given contrasting previously reported associations with CVD, including *ALR15* rs4311394 [34], *CDKN2B-AS1* rs1333049 [35] and *TNFRSF11B* rs2073618 [36].

We hypothesized that genetic variation in these candidate SNPs may be associated with incident CHD events and all-cause mortality in NI men from the PRIME study. Associations between SNPs and incident CHD events and all-cause mortality were evaluated over a 14–20 year follow-up period beginning at rescreen in 2001–2003 and ending in April 2015 for CHD events and in November 2020 for all-cause mortality.

## Methods and materials

### PRIME study participants

The PRIME study is a prospective cohort study to determine risk factors associated with incident CHD in men from NI and France [15]. Details on recruitment and examination in the PRIME study are published elsewhere [15]. In brief, there were a total of 2,745 men, aged between 50 and 60 years, initially recruited in NI between 1991 and 1994. Sampling was aimed at appropriate representation of the local population’s socioeconomic status and participation was voluntary [37]. The surviving men in the NI PRIME study were invited to attend for rescreening between 2001 and 2003. Rescreening assessment included questionnaires on socio-economic status, occupation, physical activity, tobacco/alcohol consumption and education. Weight, height, blood pressure and ECG were measured, and a blood sample for lipid and clotting profile, antioxidants, and DNA taken [15]. Approval was obtained from the Research Ethics Committee of the Faculty of Medicine, Queen’s University Belfast, and the Office for Research Ethics Committees Northern Ireland (reference number 06/NIR02/107). All men provided informed, written consent prior to participation.

### Follow-up

The study commenced on the day each of the 2,010 men attended for rescreen examination (2001–2003). The men were followed up by postal questionnaires circulated in 2007, 2011 and 2015 and asked to complete a clinical event questionnaire. Self-reported information on any potential CHD events, provided as responses to the postal questionnaires or phone contacts, was subject to extensive validation using information from hospital and medical records. Reports on any imaging performed were retrieved. Diagnostic data for all CHD events included ECGs, biomarkers, surgical interventions, revascularisation procedures and any other treatment or special investigations. The end of follow up for incident CHD events was April 1^st^, 2015. Incident CHD events included myocardial infarction, defined by one of the following sets of conditions: (a) new diagnostic Q-wave or other fresh typical electrocardiographic signs of necrosis; (b) typical or atypical pain symptoms and new (or increased) ischaemia and myocardial marker levels higher than twice the upper limit and (c) post-mortem evidence of fresh myocardial infarction or thrombosis. Incident CHD events also included coronary artery bypass grafting or percutaneous coronary intervention [38]. Previous evidence of a CVD event did not preclude participation but men with evidence of prevalent CVD at rescreening were excluded from the incident CHD study. Accurate information on a history of CHD was available from the main study database as this was the primary outcome under investigation in the PRIME study, with men having already been under observation for 10 years prior to re-screening. All the relevant information for each fatal or non-fatal coronary event was examined by a validation committee that included clinical consultants who specialised in coronary disease and were independent of the researchers in the study.

Information on deaths was obtained from the Business Services Organization (BSO), which is an agency of the Department of Health in Northern Ireland. The BSO uses death registration information supplied by the NI Register General Office and other sources to link data on all deaths in NI to the master patient index. The BSO provides six monthly updates on the deaths of participants in the cohort who are flagged on their system [39]. The end of follow-up date for mortality was November 30^th^, 2020.

### Genotyping

DNA was extracted using the salting-out method from peripheral leucocytes [25]. Genotyping was carried out at the genomic core facility at Queen’s University Belfast using a commercially available Sequenom MassARRAY iPLEX platform (Sequenom Inc., San Diego, Ca). The majority of the genetic information was obtained from blood samples taken at rescreen. If a blood sample was unavailable from rescreen, residual blood from baseline samples was used for genotyping in a small number of cases [39].

### Potential predictors of CHD

Smoking status was characterised as ‘current smoker’, ‘former smoker’ or ‘never smoked’, from questionnaire responses provided at rescreen. A history of diabetes or pre-diabetes was by self-report. Body mass index (BMI) was calculated using rescreen height and weight, and blood pressure was measured. Measurements of plasma cholesterol, HDL-cholesterol and triglycerides were determined from blood samples collected at rescreen. Some baseline information was used including alcohol intake divided into five categories– 0, 1–128, 129–265, 266–441 and > 441 ml/week. Socio-economic status was summarized as low, medium, or high, by summary variables based on vehicle ownership, number of toilets in household and home ownership status [40]. Physical activity was characterized as metabolic equivalent tasks (METs) per week [41].

### Statistical methods

Potential predictor variables were summarized on the basis of CHD events and all-cause mortality for the cohort of men who attended rescreen. Physical activity was skewed and, therefore, square root transformed before analysis. Continuous variables were compared by independent sample z test and categorical variables by chi-square test. SNP minor allele frequency (MAF) was recorded as 0, 1 or 2.

For CHD analysis, the study period ranged from date of rescreen to 1^st^ April 2015, as this was the latest date the medical committee had reviewed CHD outcomes. Men with prevalent CHD at rescreen were excluded from analysis of CHD outcomes only. For all-cause mortality, the study period was from rescreen date until 30^th^ November 2020. Cox’s proportional hazards analysis was used to estimate hazard ratios for CHD events and all-cause mortality in separate analyses, based on MAF. MAF was included as a continuous variable–giving a hazard ratio for CHD or all-cause mortality per additional minor allele. Time to CHD event was from rescreen to date of first recorded CHD event and for all-cause mortality from rescreen to date of death.

Initially an unadjusted model was evaluated, containing only MAF. An adjusted model included the additional recognised risk factors age, BMI (kg/m^2^), height (cm), systolic blood pressure measurement (mm/Hg), plasma cholesterol, HDL-cholesterol and triglyceride levels (mmol/L), physical activity (METs/week), alcohol consumption (none, 1–128, 129–265, 266–441 and > 441 mL/week), smoking status (current smoker, former smoker, never smoked), history of diabetes or prediabetes (yes, no) and socio-economic status (low, medium or high).

In addition, hazard ratios for all-cause mortality were calculated using Cox Proportional hazard analysis with MAF as a categorical variable (1 minor allele vs 0 minor allele and 2 minor alleles vs 0 minor allele comparisons). Kaplan-Meier survival curves were used to plot the cumulative incidence of all-cause mortality by the number of minor alleles for those that were significantly associated with all-cause mortality in this analysis.

The validity of the proportional hazards assumption in the Cox models was assessed by adding a time-dependent covariate (representing the interaction between genotype and time) to each model.

Statistical analysis was performed using IBM SPSS for Windows version 26 (IBM Corp, Armonk, NY), with a significance threshold p< 0.05.

## Results

### Coronary heart disease events

Of the 2,745 original PRIME participants from Northern Ireland, 2,010 attended for rescreen; 324 had CVD at entry and hence were excluded from the time to CHD event analysis. Of the remaining 1,686 men free from CVD at rescreen, 177 (10.5%) had an incident CHD event during the follow-up period to 1^st^ April 2015. Therefore, there were 1,509 controls. Potential predictor variables for CHD are summarised in Table 1. There was a significant difference (p <0.05) between groups for mean height, with those who experienced a CHD event tending to be shorter (171.7 cm versus 173.0 cm), and more likely to smoke (26.6% versus 18.2%). There was no significant difference in other recognised risk factors between those who experienced a CHD event and those that did not.

**Table 1:** Summary characteristics for coronary heart disease outcomes and all-cause mortality for 2,010 Northern Ireland men who attended rescreen, excluding 324 men with pre-existing cardiovascular disease for CHD event analysis only.

Parameter	CHD event			All-cause mortality		
	No (n = 1509)	Yes (n = 177)	*p* value	No (n = 1189)	Yes (n = 821)	*p* value
Age (years)						
Mean (SD)	64.6 (3.2)	64.5 (3.0)	0.58	**64.1 (3.0)**	**65.7 (3.1)**	**<0.001**
BMI (kg/m^2^)						
Mean (SD)	27.4 (3.6)	27.2 (3.5)	0.67	27.4 (3.6)	27.5 (3.9)	0.26
Height (cm)						
Mean (SD)	**173.0 (6.6)**	**171.7 (6.9)**	**0.02**	172.8 (6.7)	172.3 (6.5)	0.11
SBP (mmHg)						
Mean (SD)	139.8 (19.8)	142.9 (20.2)	0.57	139.4 (19.6)	140.2 (21.1)	0.35
Total cholesterol (g/L)						
Mean (SD)	5.47 (0.97)	5.43 (1.00)	0.59	**5.42 (0.97)**	**5.28 (1.03)**	**0.003**
Total HDL-cholesterol (g/L)						
Mean (SD)	1.35 (0.36)	1.29 (0.37)	0.06	1.33 (0.33)	1.36 (0.93)	0.40
Total triglyceride (g/L)						
Mean (SD)	1.74 (1.00)	1.86 (0.99)	0.13	1.74 (0.97)	1.82 (1.16)	0.11
Physical activity (METs/week)						
Median (IQR)	87 (52–132)	92 (47–136)	0.97	84 (52–132)	90 (47–133)	0.51
Alcohol consumption (mL/week) *n* (%)						
None	588 (39.0)	76 (42.9)		**489 (41.1)**	**317 (38.6)**	
1–128	289 (19.2)	25 (14.1)		**228 (19.2)**	**136 (16.6)**	
129–265	270 (17.9)	31 (17.5)		**220 (18.5)**	**147 (17.9)**	
266–441	166 (11.0)	23 (13.0)		**129 (10.8)**	**85 (10.4)**	
>441	196 (13.0)	22 (12.4)	0.49	**123 (10.3)**	**136 (16.6)**	**0.002**
Smoking status *n* (%)						
Current smoker	**275 (18.2)**	**47 (26.6)**		**186 (15.6)**	**212 (25.8)**	
Former smoker	**654 (43.3)**	**73 (41.2)**		**514 (43.2)**	**392 (47.7)**	
Never smoked	**580 (38.4)**	**57 (32.2)**	**0.023**	**489 (41.1)**	**216 (26.3)**	**<0.001**
History diabetes *n* (%)						
Yes	91 (6.0)	12 (6.8)		**71 (6.0)**	**81 (9.9)**	
No	1418 (94.0)	165 (93.2)	0.69	**1118 (94.0)**	**739 (90.0)**	**0.001**
Socio-economic status *n* (%)						
Low	527 (34.9)	54 (30.5)		**392 (33.0)**	**320 (39.0)**	
Middle	347 (23.0)	42 (23.7)		**289 (24.3)**	**160 (19.5)**	
High	634 (42.0)	81 (45.8)	0.48	**507 (42.6)**	**341 (41.5)**	**0.006**

*BMI* body mass index, *SBP* systolic blood pressure, *HDL- cholesterol* high-density lipoprotein cholesterol, *MET* metabolic equivalent task, *SD* standard deviation, *IQR* interquartile range

### All-cause mortality

All 2,010 men who attended rescreen were included in the all-cause mortality analysis. Of these, 821 (40.8%) died in the follow-up period until 30^th^ November 2020. Therefore, there were 1,189 controls. Potential predictor variables for all-cause mortality for the 2,010 men who attended rescreen are summarised in Table 1. There were significant differences (p< 0.05) between those alive and dead in terms of mean age (years)—those who died were older (65.7 years versus 64.1 years), had lower mean total cholesterol (5.28 g/L versus 5.42 g/L), were also likely to consume higher volumes of alcohol (>441 mL/week 16.6% versus 10.3%), to have been current smokers (25.8% versus 15.6%) and have a history of diabetes or prediabetes (9.9% versus 6.0%). Those that died were also more likely to have a low socio-economic status (39.0% versus 33.0%).

### Genetic associations with incident CHD events

The validity and statistical significance of the HR estimates were in line with the Cox proportional hazards assumptions tested. Adjusted hazard ratios representing associations between incident CHD events and genotype are presented in the left column of Table 2. Unadjusted hazard ratios are provided in S1 Table in S1 File. Only two SNPs (rs1800469 and rs6564851) were significantly associated with incident CHD events in the unadjusted model and both remained significant following adjustment for potential confounding variables. The minor allele for rs1800469 in *TGFB1* was associated with a per allele increased risk of incident CHD in the adjusted analysis (HR = 1.30; CI 95% 1.02–1.65; p = 0.04). The minor allele for rs6564851 in *BCMO1* was associated with a per allele reduced risk of incident CHD in the adjusted analysis (HR = 0.76; CI 95% 0.60–0.96; p = 0.02).

**Table 2:** Adjusted hazard ratios (HR) per minor allele for CHD events and all-cause mortality.

Gene (major/minor allele)	SNP ID	SNP MAF	CHD event	All-cause mortality
			HR (95% CI); *p* value	HR (95% CI); *p* value
*IL1A* (G/T)	rs17561	0.28	0.99 (0.78–1.26); 0.94	0.90 (0.79–1.02); 0.09
*IL1A* (C/T)	rs1800587	0.27	1.01 (0.80–1.29); 0.92	0.88 (0.78–1.00); 0.05
*IL1B* (G/A)	rs16944	0.30	0.93 (0.73–1.19); 0.58	**1.18 (1.05–1.33); 0.005**
*IL1RN* (T/C)	rs4251961	0.34	0.85 (0.67–1.08); 0.18	0.94 (0.84–1.06); 0.29
*IL6* (G/C)	rs1800795	0.38	1.03 (0.82–1.30); 0.80	0.99 (0.88–1.11); 0.83
*IL6* (T/C)	rs10499563	0.21	1.04 (0.79–1.37); 0.78	1.03 (0.89–1.18); 0.72
*IL6R* (A/C)	rs2228145	0.36	1.07 (0.85–1.35); 0.58	1.07 (0.95–1.21); 0.24
*IL10* (C/A)	rs1800872	0.18	1.18 (0.90–1.56); 0.23	1.00 (0.86–1.15); 0.97
*IL17A* (A/G)	rs2275913	0.30	1.13 (0.90–1.42); 0.30	1.04 (0.93–1.18); 0.49
*IL17F* (T/C)	rs763780	0.04	1.19 (0.71–2.00); 0.50	0.87 (0.65–1.17); 0.36
*TGFB1* (C/T)	rs1800469	0.25	**1.30 (1.02–1.65); 0.04**	1.09 (0.96–1.24); 0.17
*TGFB1* (G/A)	rs1982037	0.15	0.88 (0.63–1.22); 0.44	1.12 (0.96–1.31); 0.14
*S100A8* (T/C)	rs3795391	0.11	0.77 (0.53–1.13); 0.18	1.17 (0.99–1.38); 0.06
*TLR4* (A/G)	rs4986790	0.07	0.95 (0.62–1.45); 0.79	0.82 (0.65–1.03); 0.09
*COX2* (G/C)	rs6681231	0.13	0.86 (0.62–1.20); 0.37	1.05 (0.90–1.22); 0.55
*NAT2* (A/G)	rs1495741	0.19	0.90 (0.68–1.19); 0.46	0.93 (0.80–1.07); 0.30
*VDR* (T/C)	rs731236	0.35	1.09 (0.86–1.37); 0.48	1.02 (0.90–1.15); 0.78
*VDR* (G/A)	rs1544410	0.35	1.10 (0.87–1.39); 0.41	1.03 (0.91–1.16); 0.65
*VDR* (A/C)	rs7975232	0.42	0.90 (0.72–1.13); 0.36	0.97 (0.87–1.09); 0.62
*VDR* (C/T)	rs2228570	0.35	1.13 (0.90–1.41); 0.29	1.02 (0.91–1.15); 0.70
*BCMO1* (G/T)	rs6564851	0.39	**0.76 (0.60–0.96); 0.02**	1.00 (0.89–1.13); 0.96
*BCMO1* (C/T)	rs7501331	0.01	1.05 (0.81–1.36); 0.72	1.06 (0.93–1.21); 0.39
*ARL15* (A/G)	rs4311394	0.20	1.30 (1.00–1.69); 0.05	0.92 (0.80–1.06); 0.25
*SLC23A1* (G/A)	rs33972313	0.04	1.22 (0.69–2.14); 0.50	1.02 (0.75–1.38); 0.93
*CD36* (C/T)	rs13230419	0.39	0.80 (0.63–1.02); 0.07	1.00 (0.89–1.12); 0.98
*CDKN2B-AS1* (C/G)	rs1333049	0.45	0.85 (0.68–1.06); 0.14	0.94 (0.84–1.05); 0.28
*CDKN2B-AS1* (C/G)	rs518394	0.38	0.99 (0.79–1.25); 0.94	0.96 (0.85–1.08); 0.50
*CDKN2B-AS1* (A/G)	rs1360590	0.42	1.04 (0.82–1.30); 0.76	1.04 (0.93–1.17); 0.50
*TNFRSF11B* (C/G)	rs2073618	0.39	0.94 (0.75–1.19); 0.63	0.90 (0.80–1.02); 0.09
*TNFRSF11B* (C/A)	rs1872426	0.39	0.95 (0.75–1.19); 0.63	0.91 (0.81–1.02); 0.10
*TNFSF11* (A/G)	rs2277438	0.15	0.91 (0.67–1.24); 0.57	0.99 (0.85–1.15); 0.86
*GLT6D1* (C/G)	rs1537415	0.35	1.04 (0.82–1.32); 0.77	1.05 (0.93–1.18); 0.42
*FN1* (G/A)	rs33996776	0.26	1.22 (0.96–1.54); 0.10	1.04 (0.92–1.18); 0.52

The Cox Proportional HRs are adjusted for age (years), BMI (kg/m2), SBP (mmHg), total cholesterol (g/L), total HDL-cholesterol (g/L), total triglyceride (g/L), physical activity (METs/week), alcohol consumption (mL/week), smoking status, previous history of diabetes or pre-diabetes and socio-economic status. HR hazard ratio, CI confidence intervals, MAF Minor Allele Frequency

### Genetic associations with all-cause mortality

Adjusted hazard ratios representing associations between all-cause mortality and genotype are presented in the right column of Table 2. Unadjusted hazard ratios are provided in S1 Table in S1 File. Only one SNP (rs16944, *IL1B*) was significantly associated with all-cause mortality in both unadjusted and adjusted models (with a per allele HR = 1.18; CI 95% 1.05–1.33; p = 0.005 in the adjusted model).

**Fig 1:**
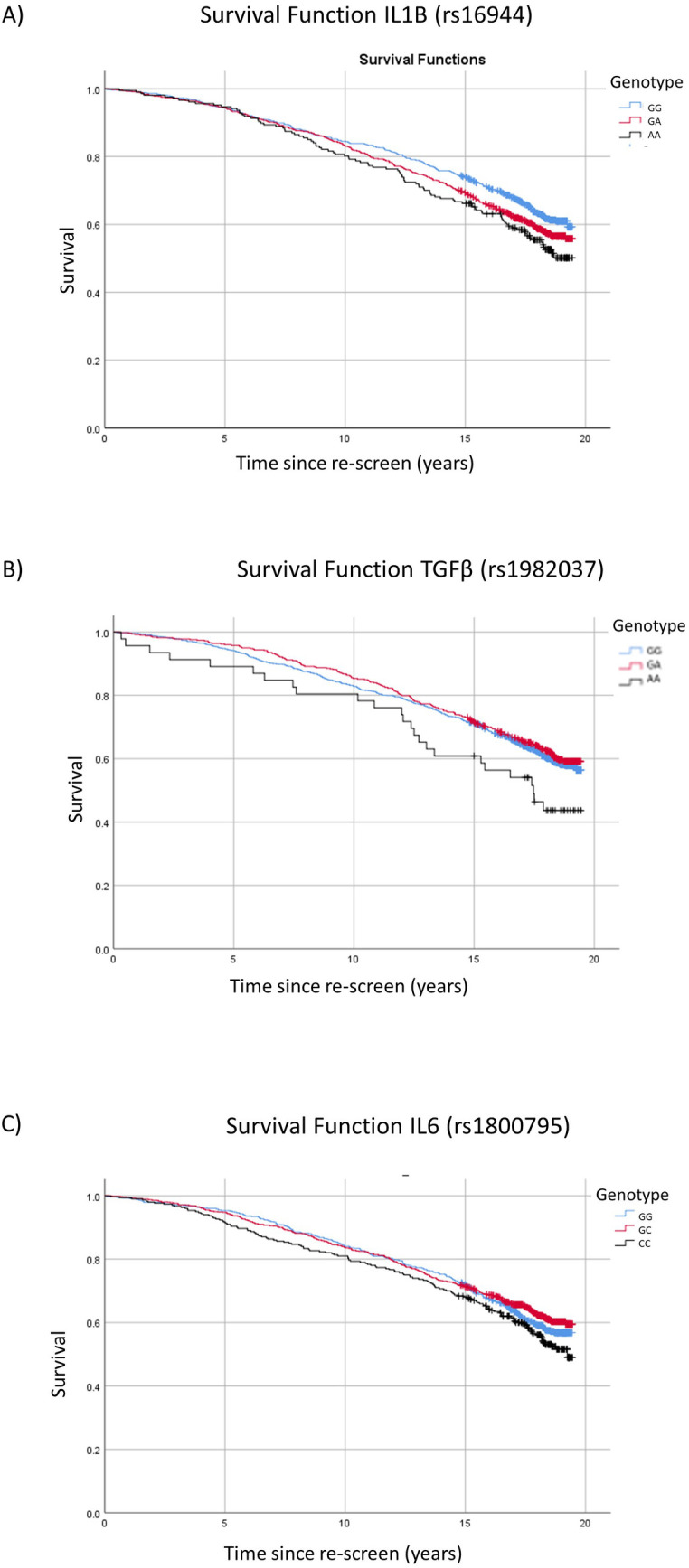
Kaplan-Meier survival analysis of all-cause mortality for significantly associated single nucleotide polymorphisms. A) *IL1B* rs16944 B) *TGFB* rs1982037 C) *IL6* rs1800795.

In additional all-cause mortality analyses that considered the number of minor alleles as a categorical variable, three SNPs were significantly associated with all-cause mortality (Table 3). Unadjusted hazard ratios are provided in S2 Table in S1 File. Compared to no copies of the minor allele for *IL1B* rs16944, one copy was associated with an increased all-cause mortality risk (HR = 1.18; CI 95% 0.99–1.41; p = 0.06), with two copies increasing the risk further (HR = 1.43; CI 95% 1.11–1.84; p = 0.005). Two copies of the minor allele of rs1982037 in *TGFB1* was also significantly associated with all-cause mortality (HR = 1.56; CI 95% 1.00–2.42; p = 0.05) compared with no copies. A single copy of the minor allele for *IL6* rs1800795 was associated with reduced all-cause mortality in the adjusted analysis (HR = 0.82; CI 95% 0.68–0.98; p = 0.03) compared to no copies, although this was not significant for two copies of the minor allele (HR = 1.06; CI 95% 0.84–1.34; p = 0.63) compared to no copies. Kaplan-Meier survival curves for significantly associated SNPs for 0, 1 or 2 minor allele copies are presented in Fig 1.

**Table 3:** Adjusted all-cause mortality hazard ratios (HR) categorical analysis.

Gene (major/minor allele)	SNP ID	All-cause mortality	
		HR (95% CI); *p* value adjusted	
		1 copy minor allele	2 copies minor allele
*IL1A* (G/T)	rs17561	0.93 (0.79–1.10); 0.41	0.77 (0.57–1.03); 0.08
*IL1A* (C/T)	rs1800587	0.88 (0.74–1.04); 0.14	0.78 (0.58–1.04); 0.09
*IL1B* (G/A)	rs16944	1.18 (0.99–1.41); 0.06	**1.43 (1.11–1.84); 0.01**
*IL1RN* (T/C)	rs4251961	1.11 (0.93–1.32); 0.24	0.75 (0.57–0.98); 0.04
*IL6* (G/C)	rs1800795	**0.82 (0.68–0.98); 0.03**	1.06 (0.84–1.34); 0.63
*IL6* (T/C)	rs10499563	0.96 (0.81–1.14); 0.64	1.25 (0.88–1.78); 0.22
*IL6R* (A/C)	rs2228145	1.03 (0.86–1.23); 0.76	1.22 (0.96–1.55); 0.11
*IL10* (C/A)	rs1800872	1.01 (0.85–1.20); 0.91	0.94 (0.61–1.46); 0.79
*IL17A* (A/G)	rs2275913	1.00 (0.83–1.19); 0.96	1.13 (0.88–1.45); 0.33
*IL17F* (T/C)	rs763780	0.96 (0.70–1.30); 0.77	-
*TGFB1* (C/T)	rs1800469	1.09 (0.92–1.30); 0.32	1.25 (0.93–1.68); 0.13
*TGFB1* (G/A)	rs1982037	1.03 (0.86–1.24); 0.72	**1.56 (1.00–2.42); 0.05**
*S100A8* (T/C)	rs3795391	1.18 (0.97–1.42); 0.10	1.28 (0.70–2.34); 0.43
*TLR4* (A/G)	rs4986790	0.84 (0.66–1.07); 0.16	0.25 (0.04–1.82); 0.17
*COX2* (G/C)	rs6681231	1.05 (0.87–1.27); 0.60	0.98 (0.61–1.57); 0.93
*NAT2* (A/G)	rs1495741	0.99 (0.83–1.18); 0.93	0.70 (0.45–1.10); 0.13
*VDR* (T/C)	rs731236	1.03 (0.86–1.22); 0.77	1.04 (0.80–1.34); 0.77
*VDR* (G/A)	rs1544410	1.02 (0.85–1.21); 0.87	1.07 (0.83–1.37); 0.60
*VDR* (A/C)	rs7975232	1.07 (0.89–1.30); 0.47	0.92 (0.73–1.17); 0.50
*VDR* (C/T)	rs2228570	1.11 (0.93–1.33); 0.25	1.05 (0.83–1.34); 0.67
*BCMO1* (G/T)	rs6564851	0.89 (0.74–1.07); 0.20	1.04 (0.82–1.30); 0.77
*BCMO1* (C/T)	rs7501331	0.98 (0.83–1.17); 0.84	1.38 (1.00–1.91); 0.05
*ARL15* (A/G)	rs4311394	0.90 (0.75–1.07); 0.23	0.92 (0.63–1.34); 0.66
*SLC23A1* (G/A)	rs33972313	1.00 (0.73–1.36); 0.99	-
*CD36* (C/T)	rs13230419	0.93 (0.78–1.13); 0.47	1.02 (0.80–1.28); 0.90
*CDKN2B-AS1* (C/G)	rs1333049	1.04 (0.85–1.26); 0.72	0.87 (0.69–1.10); 0.26
*CDKN2B-AS1* (C/G)	rs518394	1.07 (0.89–1.28); 0.49	0.85 (0.66–1.10); 0.22
*CDKN2B-AS1* (A/G)	rs1360590	1.02 (0.84–1.24); 0.85	1.11 (0.88–1.40); 0.39
*TNFRSF11B* (C/G)	rs2073618	0.99 (0.83–1.19); 0.93	0.80 (0.62–1.02); 0.07
*TNFRSF11B* (C/A)	rs1872426	0.99 (0.83–1.19); 0.92	0.80 (0.62–1.02); 0.07
*TNFSF11* (A/G)	rs2277438	0.96 (0.80–1.16); 0.69	1.18 (0.76–1.83); 0.47
*GLT6D1* (C/G)	rs1537415	1.17 (0.98–1.40); 0.09	1.05 (0.81–1.35); 0.74
*FN1* (G/A)	rs33996776	1.03 (0.87–1.23); 0.71	1.08 (0.80–1.46); 0.60

The Cox Proportional HRs are adjusted for age (years), BMI (kg/m^2^), SBP (mmHg), total cholesterol (g/L), Total HDL-cholesterol (g/L), total triglyceride (g/L), physical activity (METs/week), alcohol consumption (mL/week), smoking status, previous history of diabetes or pre-diabetes and socio-economic status. *HR* hazard ratio, *CI* confidence intervals.

### Testing the proportional hazards assumption

Time-dependent covariates added to the models in Tables 2 and 3 showed that the number attaining significance was in keeping with the number that would be expected by chance alone.

## Discussion

This study evaluated associations between 33 SNPs and incident coronary heart disease events and all-cause mortality in 2,010 men from Northern Ireland enrolled in the PRIME study. We observed a reduction in risk associated with the minor allele of rs6564851 in *BCMO1* and incident coronary events, in contrast to the observed increased risk associated with the minor allele of *TGFB* rs1800469. In addition, there was a significant association between all-cause mortality and *IL1B* rs16944. Of note, we failed to replicate some of the previously reported associations with CVD events (*IL6* rs1800795, *IL6* rs228145, *IL10* rs1800872, *IL17A* rs2275913, *VDR* rs2228570, *ARL15* rs4311394, *CDKN2B-AS1* rs1333049, *TNFRSF11B* rs2073618) [18–36].

### Association between *TGFB1* rs1800469 and CHD events

Transforming growth factor beta (TGF-β) is a cytokine family that exists as three isoforms TGF-β1, TGF-β2 and TGF-β3, encoded by genes *TGFB1*, *TGFB2* and *TGFB3* respectively [42]. TGF-β influences several cellular processes [43, 44] and has been implicated in several diseases including CVD. TGF-β has been reported as a protective cytokine against atherosclerosis [45], acting to promote plaque stabilisation [46] and reduce endothelial adhesion by T lymphocytes [47] and neutrophils [48, 49]. TGF-β has been implicated both in early atherosclerotic disease and in disease progression [49] and increases inflammation and collagen dependent stiffening of vessel walls [50, 51]. Lower TGF-β levels have been reported in patients with severe atherosclerosis [52]. In contrast, higher levels of TGF-β have been associated with increased myocardial fibrosis and worse outcomes for heart failure [53, 54].

The minor allele of the *TGFB1* promotor SNP rs1800469 is present in approximately 30% of Caucasians [55]. Twin studies have shown plasma concentrations for TGF-β1 to be twice as high for those homozygous for the minor allele compared to the homozygous wild-type genotype [56]. This is thought to result from preferential binding of the activator protein 1 to the major allele, down regulating TGFB1 promotor expression and decreasing levels of TGF-β1 [57].

In the present study, the minor allele of *TGFB1* rs1800469 was associated with an increased risk of incident coronary events (HR = 1.30; CI 95% 1.02–1.65; p 0.04), supporting findings from a recent meta-analysis that evaluated associations between rs1800496 and myocardial infarction (MI), identifying significant associations between homozygous and heterozygous minor allele genotypes and increased MI risk, compared to major allele homozygotes [58]. In contrast, a smaller NI case-control study comprising 563 cases and 629 controls, found no association between rs1800496 and MI [24]. A retrospective case-control study in men and women from a German population reported a lower risk of MI associated with the homozygous major allele genotype in men only (inverse of higher risk associated with the minor allele), with an odds ratio of 0.49 (CI 0.27–0.87) [59]. In contrast, the larger prospective Rotterdam study (n = 6,456) failed to detect any association between rs1800469 and MI [60]. Although an increased risk of stroke associated with the rs1800469 minor allele (HR = 1.26; CI 95% 1.06–1.49) [60] was reported in a previous meta-analysis, other studies have also reported no association between rs1800469 and ischaemic stroke [61].

### Association between *BCMO1* rs6564851 and CHD events

Vitamin A (retinol) is involved in several metabolic processes including vision [62], embryonic development [63] and gene expression [64]. Vitamin A is consumed in the diet as preformed vitamin A (65% of intake) or beta-carotene (35% of intake) [65]. The β-carotene-15, 15’-monooxygenase 1 (*BCMO1*) gene encodes an enzyme which catalyses oxidative cleavage of β-carotene into two molecules of retinol in the gastric mucosa [66, 67]. A common SNP, rs6564851, lies upstream from the *BCMO1* gene and the minor allele was significantly associated with higher plasma levels of beta-carotene attributed to reduced BCMO1 enzyme activity, resulting in reduced conversion of beta-carotene to retinol [68, 69].

The ATBC study, a prospective investigation of 29,000 men over 31 years of follow-up, found that higher plasma beta-carotene levels were associated with both lower all-cause and CVD-specific mortality [70]. Previous studies in the PRIME cohort reported inverse associations between retinol levels and CVD risk, and both beta-carotene and retinol levels inversely associated with all-cause mortality, in French and Northern Irish men over 10 years of follow-up [41]. The negative associations between plasma retinol and CVD were also demonstrated previously in a separate nested case-control study of PRIME participants [71].

The results of the present study demonstrate a novel association between rs6564851 and a reduced risk of incident CHD events (HR = 0.76; CI 95% 0.60–0.96; p 0.02). A small nested case-control study of Han Chinese participants with dyslipidaemia found no association between rs65644851 and coronary heart disease, although the authors raised concerns about their findings as their genotyping failed to meet a Hardy-Weinberg equilibrium quality control metric [72]. Rs6564851 has also previously been associated with higher plasma HDL cholesterol levels [73], a reported CVD protective factor [74]. While this may represent a mechanism by which rs6564851 reduces CVD risk, some genetic mechanisms that raise HDL cholesterol have not necessarily led to associations with lower CVD risk [75]. Regardless, this does not explain the effect observed in this study as HDL-cholesterol levels were adjusted for in the Cox regression model. This suggests HDL-cholesterol may act independently on CVD risk from the effect exerted by rs6564851.

The potential mechanism underlying the association between rs6564851 and incident coronary events may lie beyond traditional CVD risk factors. Yabuta and colleagues investigated the effects of daily beta-carotene supplementation by rs6564851 genotype on telomere length in buccal cells [76]. Their findings suggested homozygous individuals for the minor allele were more likely to exhibit lower levels of telomere shortening following daily beta-carotene intake. While the authors acknowledged the limited sample size of 70 Japanese participants, their findings warrant further investigation of the effects of beta-carotene supplementation and rs6564851 genotype on the mitotic aging of cells.

### Association of *IL1B* rs16944 and all-cause mortality

Interleukin 1-beta (IL-1β) is a pro-inflammatory cytokine, implicated in multiple disease states, including inflammation and pain [77–79] that hinders the functionality of the myocardium [80]. The *IL1B* promoter SNP rs16944 has been widely studied with reported associations with many diseases including lung cancer [81], rheumatoid arthritis [82] and myocardial ischaemia [83]. The only SNP significantly associated with all-cause mortality in the present study was *IL1B* rs16944, associated with an 18% increased risk of death per minor allele in adjusted models (HR = 1.18; CI 95% 1.05–1.33; p = 0.005).

A case-control study by Jiménez-Sousa et al. also reported increased sepsis related mortality with rs16944 [84]. Their study evaluated post-cardiac or abdominal surgery patients who developed systemic inflammatory response syndrome post-surgery. Despite a limited sample size (205 cases, 262 controls), the likelihood of mortality associated with rs16944 more than doubled (OR = 2.67; CI 95% 1.07–4.97; p = 0.04), highlighting the significant influence this SNP may have on sepsis-related mortality. In contrast, the *IL1B* rs16944 has also been previously reported in association with lower risk of both MI and stroke in patients with early disease onset [85], indicating the increased risk of death observed in this study may represent non-CHD related death.

### Strengths and limitations of the current study

There are several strengths to this study including the reasonable study size (n = 2,010) and large number of lifestyle, anthropometric and biochemical variables reported for each participant enabling adjustment for well-recognized CVD risk factors. Participants were of similar age and ethnicity and only a relatively small percentage were lost to follow-up. Only those free from CVD at study entry were included in the CHD time to event analysis, making this a true prospective study. There was long follow-up of participants for over 10 years for CHD events and over 15 years for all-cause mortality. The selection of participants was representative of the socio-economic make up of Northern Ireland, adding to the relevance of the results in a population context, where CVD prevalence is high [2].

However, there were also several limitations which may have limited our ability to detect previously reported associations. To minimise sampling bias, the study period defined began at rescreen in 2001–2003 –over 10 years after the original PRIME participants were recruited. This raises several issues including potential survival bias as those who died at a younger age were excluded from our analysis. Men with CVD prior to rescreen were also excluded from the incident CHD analysis, meaning high risk individuals with earlier onset CVD may not have been captured within the study time-period. There is a concern that this study period may have included men at an older age than many experience their first CHD event, with participant ages ranging between 58 and 74 years. Recent research has also suggested there is a decreased incidence of MI in the 50 to 70 years age group compared to <50 years and >70 years [86]. Despite this, our study observed 177 CHD events in 1,686 men over approximately 10 years of follow-up (10.5% participants experiencing a CHD event) compared to 169 CHD events in 2,390 NI men in the initial baseline PRIME study period over 10 years of follow-up (7.1%), suggesting our study did sufficiently capture CHD events. Another potential issue of concern was that only men who attended follow-up and remained as participants in the PRIME study were eligible for inclusion in this study–these men may have been more healthy or ‘health aware’ than those who discontinued participation in the study. Our study only included men and participants were predominantly white, limiting the generalisability of the results at a population and global level. While the adjusted model included 12 well-recognised CVD risk factors, it is possible there may have been residual confounding by variables not considered in our analysis.

Finally, for some SNPs, the MAF was low in the study population. For example, the homozygous minor allele for *SLC23A1* rs33972313 was present in only two participants. The low MAF of some SNPs meant that our study was underpowered to detect association, should it exist. While many of the SNPs included in this study were selected on the basis of a prior hypothesis of association with CVD events or all-cause mortality, a Bonferroni correction for multiple testing indicates none would retain their significance at the P< 0.05/33 = 0.0015 level.

## Conclusion

In conclusion, we have shown associations between *TGFB1* rs1800469 and *BCMO1* rs6564851 with incident coronary events in male participants of the PRIME study. In addition, *IL1B* rs16944 was also significantly associated with all-cause mortality. The findings of this study add to the existing literature of disease association with these SNPs and increase the knowledge base of the genetic risk associated with CHD and all-cause mortality. In the future SNP associations, such as those studied here, may be used to identify individuals and families at higher CVD risk, allowing more targeted education on modifiable risk factors, in particular for those in whom aggressive treatment is required, and perhaps enabling the improved prediction of further event outcomes. Additional research is needed to support the associations reported here, particularly for the association between incident coronary events and *BCMO1* rs6564851, as to the best of our knowledge, this is a novel finding.

## Supporting information

S1 File Unadjusted hazard ratios for coronary heart disease events and all-cause mortality. (DOCX)
