# Supplementary material for: Association of TGFB1 rs1800469 and BCMO1 rs6564851 with coronary heart disease and IL1B rs16944 with all-cause mortality in men from the Northern Ireland PRIME study
Source: PLoS One. 2022 Aug 22;17(8):e0273333. doi: 10.1371/journal.pone.0273333 (PMC9394803; doi:10.1371/journal.pone.0273333)
Supplement: S1 File — (DOCX) [file pone.0273333.s001.docx]

**Supplementary Table S1**. Unadjusted hazard ratios (HR) per minor allele for CHD events and all-cause mortality

| **Gene (major/minor allele)** | **SNP ID** | **SNP MAF** | | | **CHD event** | **All-cause mortality** |
| --- | --- | --- | --- | --- | --- | --- |
|  |  | **0 copies** | **1 copy** | **2 copies** | **HR (95% CI); *p* value** | **HR (95% CI); *p* value** |
| *IL1A* (G/T) | rs17561 | 858 | 757 | 177 | 0.98 (0.77-1.25); 0.89 | 0.91 (0.82-1.02); 0.12 |
| *IL1A* (C/T) | rs1800587 | 867 | 752 | 174 | 1.00 (0.79-1.28); 0.89 | 0.89 (0.80-1.00); 0.05 |
| *IL1B* (G/A) | rs16944 | 781 | 802 | 207 | 0.96 (0.75-1.21); 0.71 | 1.16 (1.04-1.29); 0.01 |
| *IL1RN* (T/C) | rs4251961 | 693 | 851 | 250 | 0.85 (0.67-1.08); 0.18 | 0.90 (0.81-1.00); 0.06 |
| *IL6* (G/C) | rs1800795 | 577 | 918 | 300 | 1.05 (0.84-1.32); 0.67 | 1.05 (0.95-1.17); 0.35 |
| *IL6* (T/C) | rs10499563 | 1032 | 662 | 82 | 1.03 (0.79-1.35); 0.84 | 0.97 (0.85-1.10); 0.59 |
| *IL6R* (A/C) | rs2228145 | 630 | 859 | 295 | 1.04 (0.83-1.31); 0.71 | 1.08 (0.97-1.20); 0.15 |
| *IL10* (C/A) | rs1800872 | 1132 | 596 | 72 | 1.15 (0.88-1.51); 0.31 | 0.94 (0.83-1.08); 0.38 |
| *IL17A* (A/G) | rs2275913 | 728 | 739 | 225 | 1.16 (0.92-1.45); 0.20 | 1.05 (0.94-1.17); 0.37 |
| *IL17F* (T/C) | rs763780 | 1640 | 150 | 3 | 1.24 (0.74-2.08); 0.41 | 0.91 (0.70-1.19); 0.50 |
| *TGFB1* (C/T) | rs1800469 | 922 | 735 | 137 | 1.29 (1.01-1.64); 0.04 | 1.07 (0.95-1.20); 0.27 |
| *TGFB1* (G/A) | rs1982037 | 1247 | 498 | 46 | 0.88 (0.63-1.21); 0.42 | 1.03 (0.90-1.19); 0.65 |
| *S100A8* (T/C) | rs3795391 | 1382 | 388 | 28 | 0.80 (0.55-1.16); 0.24 | 1.07 (0.92-1.25); 0.37 |
| *TLR4* (A/G) | rs4986790 | 1501 | 261 | 13 | 0.94 (0.62-1.43); 0.77 | 0.88 (0.72-1.07); 0.20 |
| *COX2* (G/C) | rs6681231 | 1311 | 427 | 54 | 0.89 (0.64-1.23); 0.49 | 1.08 (0.94-1.24); 0.26 |
| *NAT2* (A/G) | rs1495741 | 1104 | 606 | 81 | 0.92 (0.70-1.22); 0.56 | 0.94 (0.82-1.06); 0.30 |
| *VDR* (T/C) | rs731236 | 654 | 884 | 256 | 1.08 (0.86-1.36); 0.51 | 0.99 (0.89-1.11); 0.91 |
| *VDR* (G/A) | rs1544410 | 641 | 874 | 274 | 1.09 (0.87-1.37); 0.46 | 0.99 (0.89-1.11); 0.91 |
| *VDR* (A/C) | rs7975232 | 498 | 898 | 395 | 0.92 (0.73-1.15); 0.47 | 1.00 (0.90-1.10); 0.93 |
| *VDR* (C/T) | rs2228570 | 668 | 846 | 283 | 1.13 (0.89-1.40); 0.34 | 1.00 (0.91-1.12); 0.92 |
| *BCMO1* (G/T) | rs6564851 | 576 | 880 | 337 | 0.76 (0.60-0.95); 0.02 | 1.01 (0.91-1.12); 0.90 |
| *BCMO1* (C/T) | rs7501331 | 53 | 32 | 3 | 1.02 (0.79-1.32); 0.87 | 1.06 (0.94-1.19); 0.36 |
| *ARL15* (A/G) | rs4311394 | 996 | 614 | 95 | 1.28 (0.98-1.65); 0.07 | 0.93 (0.82-1.06); 0.26 |
| *SLC23A1* (G/A) | rs33972313 | 1661 | 136 | 2 | 1.11 (0.63-1.94); 0.72 | 0.90 (0.68-1.18); 0.44 |
| *CD36* (C/T) | rs13230419 | 552 | 890 | 340 | 0.85 (0.67-1.07); 0.16 | 0.99 (0.89-1.10); 0.88 |
| *CDKN2B-AS1* (C/G) | rs1333049 | 442 | 900 | 449 | 0.83 (0.66-1.04); 0.10 | 0.93 (0.84-1.03); 0.19 |
| *CDKN2B-AS1* (C/G) | rs518394 | 567 | 905 | 301 | 0.98 (0.78-1.24); 0.88 | 0.92 (0.82-1.02); 0.11 |
| *CDKN2B-AS1* (A/G) | rs1360590 | 493 | 911 | 385 | 1.06 (0.84-1.33); 0.63 | 1.07 (0.96-1.19); 0.21 |
| *TNFRSF11B* (C/G) | rs2073618 | 552 | 917 | 331 | 0.97 (0.77-1.22); 0.77 | 0.94 (0.84-1.04); 0.21 |
| *TNFRSF11B* (C/A) | rs1872426 | 550 | 906 | 330 | 0.96 (0.77-1.21); 0.76 | 0.93 (0.84-1.04); 0.20 |
| *TNFSF11* (A/G) | rs2277438 | 1234 | 502 | 54 | 0.94 (0.69-1.27); 0.67 | 1.07 (0.93-1.23); 0.33 |
| *GLT6D1* (C/G) | rs1537415 | 632 | 907 | 254 | 1.07 (0.85-1.35); 0.57 | 1.05 (0.94-1.17); 0.39 |
| *FN1* (G/A) | rs33996776 | 869 | 729 | 160 | 1.19 (0.94-1.50); 0.15 | 0.95 (0.85-1.06); 0.35 |
| *HR* hazard ratio, *CI* confidence intervals, *MAF* Minor Allele Frequency | | | | | | |

**Supplementary Table S2.** Unadjusted all-cause mortality hazard ratios (HR) categorical analysis.

| **Gene (major/minor allele)** | **SNP ID** | **All-cause mortality HR (95% CI); *p* value** | |
| --- | --- | --- | --- |
|  |  | **1 copy minor allele** | **2 copies minor allele** |
| *IL1A* (G/T) | rs17561 | 0.91 (0.78-1.06); 0.24 | 0.84 (0.65-1.09); 0.19 |
| *IL1A* (C/T) | rs1800587 | 0.87 (0.74-1.01); 0.07 | 0.82 (0.63-1.07); 0.15 |
| *IL1B* (G/A) | rs16944 | 1.16 (0.99-1.36); 0.06 | **1.34 (1.06-1.69); 0.01** |
| *IL1RN* (T/C) | rs4251961 | 1.03 (0.88-1.20); 0.71 | 0.72 (0.56-0.92); 0.01 |
| *IL6* (G/C) | rs1800795 | 0.92 (0.78-1.09); 0.34 | 1.16 (0.94-1.43); 0.16 |
| *IL6* (T/C) | rs10499563 | 0.93 (0.80-1.09); 0.38 | 1.03 (0.73-1.46); 0.88 |
| *IL6R* (A/C) | rs2228145 | 0.98 (0.83-1.15); 0.78 | 1.21 (0.98-1.50); 0.07 |
| *IL10* (C/A) | rs1800872 | 0.95 (0.81-1.11); 0.48 | 0.89 (0.60-1.31); 0.55 |
| *IL17A* (A/G) | rs2275913 | 0.99 (0.84-1.16); 0.89 | 1.15 (0.92-1.45); 0.22 |
| *IL17F* (T/C) | rs763780 | 0.96 (0.73-1.25); 0.75 | - |
| *TGFB1* (C/T) | rs1800469 | 0.99 (0.85-1.15); 0.87 | 1.28 (0.98-1.66); 0.07 |
| *TGFB1* (G/A) | rs1982037 | 0.94 (0.80-1.11); 0.48 | **1.52 (1.02-2.27); 0.04** |
| *S100A8* (T/C) | rs3795391 | 1.05 (0.88-1.25); 0.61 | 1.33 (0.77-2.30); 0.31 |
| *TLR4* (A/G) | rs4986790 | 0.94 (0.76-1.15); 0.53 | 0.32 (0.08-1.28); 0.11 |
| *COX2* (G/C) | rs6681231 | 1.06 (0.90-1.26); 0.48 | 1.24 (0.83-1.85); 0.30 |
| *NAT2* (A/G) | rs1495741 | 1.00 (0.86-1.17); 0.97 | 0.70 (0.47-1.05); 0.09 |
| *VDR* (T/C) | rs731236 | 1.05 (0.89-1.23); 0.59 | 0.96 (0.76-1.21); 0.70 |
| *VDR* (G/A) | rs1544410 | 1.04 (0.88-1.22); 0.67 | 0.97 (0.77-1.21); 0.76 |
| *VDR* (A/C) | rs7975232 | 1.17 (0.98-1.39); 0.08 | 0.97 (0.78-1.20); 0.78 |
| *VDR* (C/T) | rs2228570 | 1.05 (0.90-1.23); 0.54 | 0.99 (0.79-1.23); 0.90 |
| *BCMO1* (G/T) | rs6564851 | 0.91 (0.77-1.07); 0.26 | 1.04 (0.85-1.29); 0.68 |
| *BCMO1* (C/T) | rs7501331 | 1.00 (0.86-1.17); 0.96 | 1.22 (0.92-1.63); 0.17 |
| *ARL15* (A/G) | rs4311394 | 0.87 (0.74-1.02); 0.09 | 1.00 (0.72-1.38); 1.00 |
| *SLC23A1* (G/A) | rs33972313 | 0.93 (0.70-1.23); 0.61 | - |
| *CD36* (C/T) | rs13230419 | 0.91 (0.77-1.08); 0.29 | 1.01 (0.82-1.24); 0.95 |
| *CDKN2B-AS1* (C/G) | rs1333049 | 1.00 (0.84-1.20); 0.98 | 0.87 (0.71-1.07); 0.19 |
| *CDKN2B-AS1* (C/G) | rs518394 | 1.04 (0.88-1.22); 0.67 | 0.79 (0.62-1.00); 0.05 |
| *CDKN2B-AS1* (A/G) | rs1360590 | 1.12 (0.94-1.33); 0.21 | 1.14 (0.92-1.41); 0.23 |
| *TNFRSF11B* (C/G) | rs2073618 | 0.99 (0.84-1.17); 0.91 | 0.85 (0.68-1.06); 0.16 |
| *TNFRSF11B* (C/A) | rs1872426 | 0.99 (0.84-1.16); 0.86 | 0.85 (0.68-1.06); 0.16 |
| *TNFSF11* (A/G) | rs2277438 | 1.01 (0.86-1.19); 0.91 | 1.38 (0.94-2.04); 0.10 |
| *GLT6D1* (C/G) | rs1537415 | 1.17 (1.00-1.38); 0.06 | 1.03 (0.82-1.31); 0.78 |
| *FN1* (G/A) | rs33996776 | 0.95 (0.82-1.11); 0.52 | 0.89 (0.68-1.17); 0.41 |

*HR* hazard ratio, *CI* confidence intervals
